# Supplementary material for: Testis-Specific Protein Y-Encoded (TSPY) Is Required for Male Early Embryo Development in Bos taurus
Source: Int J Mol Sci. 2023 Feb 8;24(4):3349. doi: 10.3390/ijms24043349 (PMC9959854; doi:10.3390/ijms24043349)
Supplement: Supplementary file 1 [file ijms-24-03349-s001.zip › ijms-2180176-supplementary.pdf]

Supplementary Figure S1

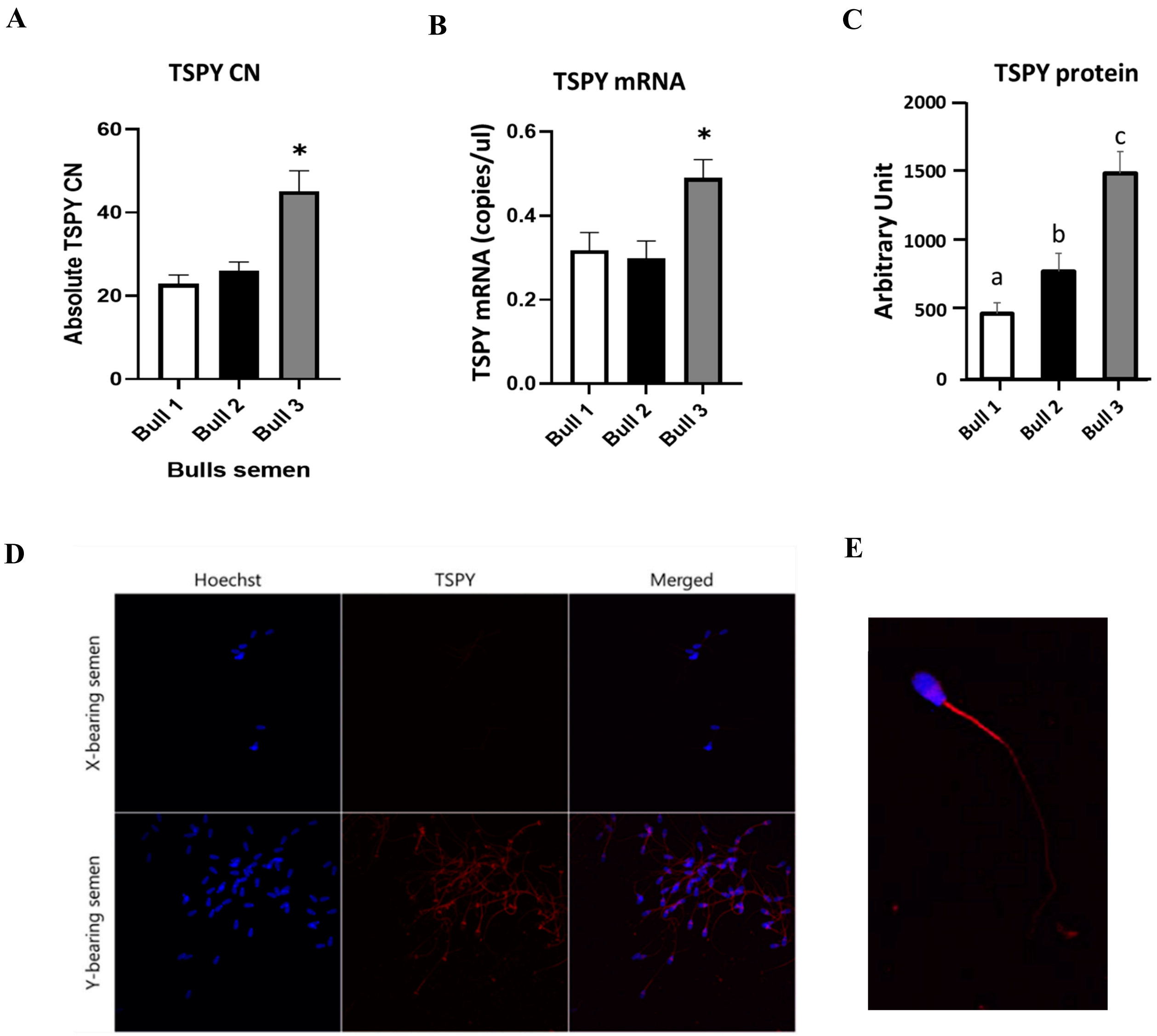

Figure S1. TSPY CN, mRNA and protein in Y-bearing semen from bull 1, 2 and 3. **A**, **B**, and **C** represent absolute TSPY CN, mRNA and protein levels respectively, in 50 Y-bearing sperm from bulls 1, 2 and 3. The experiment was conducted in 5 biological replicates of pools of 50 sperm and 2 technical replicates. The data is shown as mean ± SEM; asterisks above the column indicate significant differences $p < 0.05$. Images of TSPY protein localization of the X- and Y- bearing semen from the three bulls are shown in **D**. Hoechst, TSPY and Merged are the nucleus, TSPY protein and combined images, respectively. **E** is an enlarged image of Y-bearing sperm showing TSPY localization.
